# Chikungunya Outbreak, Cuba, July 2025

**DOI:** 10.3201/eid3207.260344

**Published:** 2026-07

**Authors:** Melissa M. Perez, Sonia Resik, Berta Maria Bello Rodriguez, Ariamys Companioni, Daniel Gonzalez, Ana Julia Benitez, Yanet Martinez, Mónica Sanchez, Mayling Alvarez, Denelsys Hernandez, Jose Raul de Armas, Aliuska Batista Serrano, Madelaine Rivera, Carilda Peña, Liannet Domínguez Ramos, Silvia Serrano, Rosario Gravier, Lorena Vazquez, Vivian Kourí, Maria G. Guzman

**Affiliations:** Pedro Kouri Tropical Medicine Institute, Havana, Cuba (M.M. Perez, S. Resik, A. Companioni, D. Gonzalez, A.J. Benitez, Y. Martinez, M. Sanchez, M. Alvarez, D. Hernandez, S. Serrano, R. Gravier, L. Vazquez, V. Kourí, M.G. Guzman); Matanzas Provincial Center for Hygiene, Epidemiology and Microbiology, Matanzas, Cuba (B.M. Bello Rodriguez); Ministerio de Salud Pública, Havana (J.R. de Armas, M. Rivera, C. Peña); Polyclinic 30th Anniversary, Perico Municipality, Matanzas (A. Batista Serrano, L. Domínguez Ramos)

**Keywords:** Chikungunya, viruses, vector-borne infections, outbreak, Cuba, ECSA genotype

## Abstract

Chikungunya transmission was confirmed in Perico, Matanzas Province, Cuba. Initial research confirmed the presence of East/Central/South African genotype related to Brazil 2025 strains in serum samples and in *Aedes aegypti* mosquito pools from transmission areas. Our findings underscore the need for surveillance and signal potential spread to other regions.

In 2004, the global epidemiology of chikungunya shifted, when the virus spread from Kenya to islands in the Indian Ocean. By 2013, transmission reached the French Caribbean and subsequently expanded throughout the Americas. In 2025, the Pan American Health Organization reported 631,720 suspected chikungunya cases, primarily in Brazil, Bolivia, Argentina, and Paraguay (*1*). Viral persistence in the Americas is driven by a combination of climatic, economic, social, demographic, and entomovirologic factors (*2*).

Cuba maintains a national dengue surveillance system for acute febrile illness (AFI) of unknown etiology. Serum samples are routinely tested for dengue IgM at local laboratories, and molecular testing by quantitative reverse transcription PCR (qRT-PCR) is conducted at the Arbovirus National Reference Laboratory of the Institute of Tropical Medicine Pedro Kouri (*3*).

On July 16, 2025, an increase in AFI cases was reported in España Republicana, Perico Municipality, Matanzas Province. Serum samples from AFI cases collected for dengue IgM detection tested negative at Perico laboratory. On July 21, the reference laboratory received 12 serum samples from patients with AFI from España Republicana and tested extracted RNA by qRT-PCR using QIAmp Viral RNA Mini Kit (QIAGEN, https://www.qiagen.com) and VIASURE multiplex test (Certest Biotec, https://www.certest.es) for dengue (DENV), Zika, chikungunya (CHIKV), Mayaro, Oropouche, and yellow fever viruses (*4*). Eight (66.7%) samples tested positive for CHIKV and 1 (8.3%) for DENV.

Confirmed chikungunya patients experienced high-grade fever lasting 48–72 hours and unresponsive to antipyretics, and severe disabling joint pain, predominantly in the hands, ankles, and back. Inflammation of the affected joints and a pruritic maculopapular rash were observed at various stages of the illness. Additional symptoms included oral lesions, vomiting, nausea, loss of appetite, diarrhea, and malaise. Median age was 46 (range 15–72) years. An equal number of male and female patients were affected. No severe or fatal cases were identified in that initial cluster.

To assess the extent of transmission, we collected acute serum samples from AFI patients in Perico (15 samples) and neighboring municipalities (39 samples) on July 23 (Table). All samples tested negative for dengue IgM. All Perico samples tested positive for CHIKV; 1 case had DENV co-infection. No CHIKV was detected in neighboring municipalities, although 2 samples were positive for DENV.

**Table:** Characteristics of samples collected from symptomatic patients during chikungunya outbreak, Matanzas Province, Cuba, 2025*

Municipality	No. tested	Epidemiologic week collected	Mean (range) days of sample collection†	CHIKV-positive			DENV-positive	
				No. (%)	Mean (range) Ct value‡		No. (%)	Mean Ct value‡
Perico	12	29	6.5 (2–11)	8 (67)	30.40 (24.25–35.03)		1 (6.6)	37.8
	15	30	1 (1–3)	15 (100)	23.05 (19.10–30.50)		1 (6.6)	39.7
Cardenas	2	28–29	6 (6–6)	0	NA		0	NA
Jovellanos	3	28–30	6 (6–8)	0	NA		1 (33.3)	39.3
Colon	27	27–30	6 (6–6)	0	NA		0	NA
Limonar	3	28	6 (6–10)	0	NA		0	NA
Matanzas city	4	28	4 (1–9)	0	NA		1 (25)	39.6
Total	66	NA	5.07 (1–9)	23 (34.8)	NA		4 (6.06)	NA

*Internal control was positive for all tested samples by quantitative reverse transcription PCR. One sample from Perico municipality tested positive for both CHIKV and DENV. CHIKV, chikungunya virus; Ct, cycle threshold; DENV, dengue virus; NA, not applicable. †Days of sample collection from illness onset. ‡Ct threshold was <40 for both CHIKV and DENV by quantitative reverse transcription PCR.

After confirming CHIKV transmission, national surveillance was intensified through active AFI case finding; standardized case definitions for suspected and confirmed cases were implemented (*5*). By epidemiologic week 52, transmission was confirmed in 15 provinces and 147 municipalities, including 49,258 suspected cases, 1,959 confirmed cases, and 46 deaths. Severe cases included neurologic complications (encephalitis, Guillain-Barré syndrome, meningoencephalitis), cardiovascular manifestations (acute myocarditis, decompensation of preexisting conditions, pulmonary thromboembolism), and perinatal or neonatal transmission.

We conducted entomologic investigations in the home of the first identified case from a town in Perico and 9 neighboring households. We collected a total of 16 *Culex quinquefasciatus* and 8 *Aedes aegypti* mosquitoes and grouped them into 3 pools: 10 engorged *Cx. quinquefasciatus,* 3 engorged *Ae. aegypti*, and 2 nonengorged *Ae. aegypti*. All pools tested positive for CHIKV by qRT-PCR. Cycle threshold cutoff was 40; values were 36.1 in the *Cx. quinquefasciatus* pool, 39.6 in the engorged *Ae. aegypti* pool, and 21.7 in the nonengorged *Ae. aegypti* pool (Appendix Table).

The detection of CHIKV in engorged mosquitoes of both species suggests the presence of the virus in the blood of residents. Detection in nonengorged *Ae. aegypti* mosquitoes supports active viral replication and ongoing transmission. We ruled out contamination, because all extraction and PCR negative controls were negative and mosquito samples were processed separately from human specimens.

For genetic characterization, we sequenced CHIKV from 2 human serum samples and 2 mosquito pools (engorged and nonengorged *Ae. aegypti*) using the MinION (Oxford Nanopore Technologies, https://nanoporetech.com), achieving >80% genome coverage. Phylogenetic analysis showed that the Cuba sequences formed a monophyletic clade, sharing a most recent common ancestor with Brazil sequences from 2021 and 2025, and clustering with sequences from Uruguay, Paraguay, and Argentina from 2023 (Figure; Appendix). We classified the virus within genotype II ECSA, consistent with strains circulating in the Americas (Chikungunya Typing Tool version 3.72 (Genome Detective, https://www.genomedetective.com). We did not detect the E1-A226V mutation associated with increased infectivity in *Ae. albopictus* (*8*). We deposited sequences in GISAID (accession nos. EPI_ISL_20294022–25).

**Figure:**
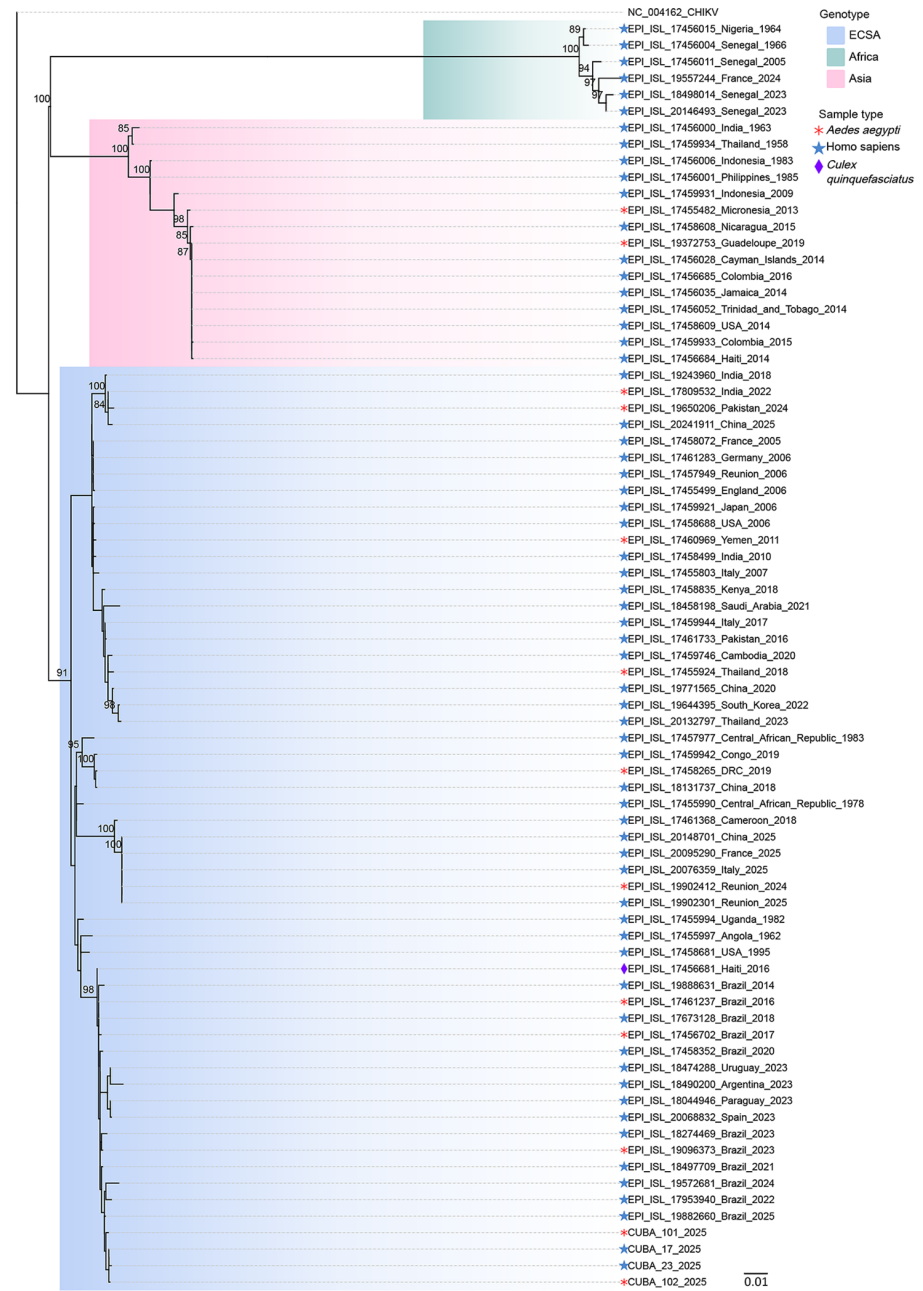
Molecular phylogenetic analysis of chikungunya virus from outbreak in Matanzas Province, Cuba, 2025. We obtained all available chikungunya virus sequences from GISAID, then filtered the sequences to ensure dataset and genetic diversity. We inferred evolutionary history by maximum-likelihood method by using the Tamura-Nei model (*6*). We applied the neighbor-joining method to a matrix of estimated pairwise distances to obtain initial trees and used a discrete gamma distribution to model evolutionary rate differences among sites. We conducted evolutionary analyses in MEGA version 6 (*7*). Numbers at branches indicate the percentage of trees in which associated taxa clustered together. Scale bar indicates number of substitutions per site. DRC, Democratic Republic of the Congo; USA, United States.

Our findings document a chikungunya outbreak in Cuba. A previous outbreak occurred in Santiago de Cuba Province in 2015 and was successfully contained; no subsequent transmission was detected (*3*). This outbreak underscores the importance of strengthening arbovirus surveillance systems. Early detection was achieved in a small town where the virus had not been previously reported, despite the presence of competent vectors and substantial travel exchange with other countries in the region.

In conclusion, chikungunya incidence might be increasing globally because of favorable climatic and environmental conditions, as well as the accumulation of susceptible populations. The Cuba outbreak highlights the need for integrated clinical, epidemiologic, virologic, and entomologic surveillance to explore additional mutations and clarify introduction pathways of arboviruses in the Americas.

Appendix Additional information about chikungunya outbreak, Cuba, 2025.
