# Supplementary material for: Chikungunya Outbreak, Cuba, July 2025
Source: Emerg Infect Dis. 2026 Jul;32(7):1222–5. doi: 10.3201/eid3207.260344 (PMC13322416; doi:10.3201/eid3207.260344)
Supplement: Appendix — Additional information about chikungunya outbreak, Cuba, 2025. [file 26-0344-Techapp-s1.pdf]

Article DOI: https://doi.org/10.3201/eid3207.260344

EID cannot ensure accessibility for supplementary materials supplied by authors. Readers who have difficulty accessing supplementary content should contact the authors for assistance.

Chikungunya Outbreak, Cuba, July 2025

Appendix

Molecular Phylogenetic Analysis

The evolutionary history was inferred by using the maximum-likelihood method based on the Tamura-Nei model. The tree with the highest log likelihood (-7105.4176) is shown. The percentage of trees in which the associated taxa clustered together is shown next to the branches. Initial tree(s) for the heuristic search were obtained by applying the neighbor-joining method to a matrix of pairwise distances estimated using the Maximum Composite Likelihood (MCL) approach. A discrete Gamma distribution was used to model evolutionary rate differences among sites (5 categories (+*G*, parameter = 0.3274)). The tree is drawn to scale, with branch lengths measured in the number of substitutions per site. The analysis involved 79 nucleotide sequences deposited in GISAID from the different outbreaks and genotypes of CHIKV that occurred in the Americas and the Caribbean region. Evolutionary analyses were conducted in MEGA6. Our study samples were labeled as CUBA sample number_2025 (EPI_ISL_20294022, EPI_ISL_20294023, EPI_ISL_20294024, EPI_ISL_20294025). Complete viral genomes were obtained from the public database GISAID. Initially, all available sequences corresponding to CHIKV were downloaded. Subsequently, the sequences were filtered to ensure the quality of the dataset. Incomplete genomes, sequences containing more than 5% ambiguous nucleotides, and duplicates derived from the same viral isolate were excluded. In order to adequately represent the genetic diversity of the virus, genomes from different geographic regions and sampling dates were selected. The presence of the closest related genomes identified through BLAST analysis was ensured. Chikungunya Virus Typing Tool from Genome Detective was used as the genotyping tool.

Appendix Table. CT values of studied mosquito pools

Mosquito species	Engorged (Y/N)	Ct value CHIKV qRT-PCR*	Nucleotidic sequence (Y/N) (sample number)
Cx. quinquefasciatus	Y	36.1	N (100)
Ae. aegypti	Y	39.6	Y (101)
Ae. aegypti	N	21.7	Y (102)

*Cutoff ≤40.
